# Plasma Aromatic L-Amino Acid Decarboxylase Activity by HPLC as a Functional Biomarker for the Diagnosis of Aromatic L-Amino Acid Decarboxylase Deficiency

**DOI:** 10.3390/metabo16070444

**Published:** 2026-06-25

**Authors:** Norashareena Mohamed Shakrin, Norzahidah Khalid, Nor Azimah Abdul Azize, Yusnita Yakob, Abdah Md. Akim, Julaina Abdul Jalil

**Affiliations:** 1 Inborn Errors of Metabolism & Genetics Unit, Nutrition, Metabolic & Cardiovascular Research Centre, Institute for Medical Research (IMR), National Institutes of Health (NIH), Ministry of Health Malaysia, Persiaran Setia Murni U13/52, Bandar Setia Alam, Shah Alam 40170, Malaysia; 2 Department of Biomedical Sciences, Faculty of Medicine & Health Sciences, Universiti Putra Malaysia (UPM), Serdang 43400, Malaysia; 3 Biochemistry Unit, Specialized Diagnostic Centre, Institute for Medical Research (IMR), National Institutes of Health (NIH), Ministry of Health Malaysia, Jalan Pahang, Kuala Lumpur 50588, Malaysia; 4 Molecular Diagnostics Unit, Specialized Diagnostic Centre, Institute for Medical Research (IMR), National Institutes of Health (NIH), Ministry of Health Malaysia, Jalan Pahang, Kuala Lumpur 50588, Malaysia

**Keywords:** Aromatic-L-amino acid decarboxylase deficiency, biomarkers, *DDC* gene, dopamine, serotonin, High-Performance Liquid Chromatography, Inborn errors of metabolism, clinical metabolomics

## Abstract

**Highlights:**

**What are the main findings?**

- A High-Performance Liquid Chromatography (HPLC)-based assay was optimized and analytically evaluated for direct measurement of plasma aromatic L-amino acid decarboxylase (AADC) activity using both L-dopa and 5-HTP as physiological substrates.
- Preliminary clinical application demonstrated reduced plasma AADC activity in genetically confirmed AADC-deficient patients.

**What are the implications of the main findings?**

- Plasma AADC enzymatic activity has potential utility as a minimally invasive functional biomarker that complements molecular genetic testing and improves diagnostic accuracy for neurotransmitter disorders.
- The established assay provides a practical tool for biochemical screening and could potentially be utilized to monitor the efficacy of emerging gene therapies and other enzyme-restoration treatments.

**Abstract:**

**Background/Objectives**: Aromatic L-amino acid decarboxylase deficiency (AADC-D; OMIM #608643) is a rare autosomal recessive neurometabolic disorder caused by pathogenic variants in the *DDC* gene, leading to impaired of monoamine neurotransmitter biosynthesis. AADC, a pyridoxal-5′-phosphate (PLP)-dependent enzyme, catalyzes the conversion of L-dopa and 5-hydroxytryptophan (5-HTP) to dopamine and serotonin, respectively. Early diagnosis remains challenging due to the limited specificity of current biochemical approaches. This study aimed to evaluate plasma AADC enzyme activity using these physiological substrates by High-Performance Liquid Chromatography (HPLC)-based method and assess its potential utility in the biochemical diagnosis of AADC deficiency. **Methods**: Plasma AADC activity was quantified using physiological substrates (L-dopa and 5-HTP) by HPLC with electrochemical and fluorescence detection. Sanger sequencing of the *DDC* gene was performed in two suspected patients to identify pathogenic variants. **Results**: Two genetically confirmed AADC-D patients demonstrated reduced enzyme activity. Using L-dopa as substrate, enzyme activity in patients was 12.4 and 26.1 pmol/min/mL, both below the published reference interval (36–129 pmol/min/mL). Using 5-HTP as substrate, enzyme activity was 1.5 and 5.1 pmol/min/mL; Patient 1 showed activity below the reference interval (2.0–7.1 pmol/min/mL), while Patient 2 demonstrated activity within the lower range of reported values. Reduced enzyme activity was consistent with the clinical features and molecular findings with identification of pathogenic variants in the *DDC* gene (c.175G>A and c.714+4A>T). **Conclusions**: Plasma AADC activity measurement demonstrates potential as a functional biochemical biomarker that augments molecular genetic testing in the biochemical evaluation of AADC deficiency. Further studies involving larger patient cohorts are required to further evaluate its diagnostic performance and broader clinical applicability.

## 1. Introduction

Aromatic L-amino acid decarboxylase deficiency (AADC-D; OMIM #608643) is a rare autosomal recessive neurometabolic disorder caused by biallelic pathogenic variants in the *DDC* gene, resulting in markedly reduced activity of the aromatic L-amino acid decarboxylase (AADC) enzyme (EC 4.1.1.28). First described in the early 1990s, this disorder disrupts the final enzymatic step in monoamine neurotransmitter biosynthesis, leading to profound impairment of dopamine and serotonin production [1,2]. The AADC enzyme, also known as DOPA decarboxylase, is a pyridoxal-5′-phosphate (PLP)-dependent enzyme that catalyzes the decarboxylation of L-3,4-dihydroxyphenylalanine (L-dopa) and 5-hydroxytryptophan (5-HTP), thereby completing the biosynthetic pathways of catecholamines and indoleamines [3,4,5,6]. Deficiency of AADC results in a characteristic metabolic phenotype characterized by reduced downstream monoamine neurotransmitters and accumulation of upstream precursors, reflecting impaired metabolic flux within these neurochemical pathways [7,8,9].

Monoamine neurotransmitters play essential roles in central nervous system development and function, regulating motor control, autonomic function, mood, and cognition. Consequently, impaired monoamine biosynthesis in AADC-D leads to severe neurological manifestations, typically presenting in early infancy [10]. Clinical features commonly include hypotonia, movement disorders such as oculogyric crises and dystonia, developmental delay, autonomic dysfunction, and feeding difficulties [11,12]. Although most patients present with severe phenotypes, clinical heterogeneity exists, with some individuals exhibiting milder or atypical manifestations that contribute to diagnostic challenges [5,13,14,15]. The overlap of clinical symptoms with other neurometabolic and neurodevelopmental disorders frequently results in delayed diagnosis, with reported mean diagnostic ages of several years after symptom onset [14].

The global incidence of AADC-D remains uncertain due to underdiagnosis and limited awareness; however, epidemiological studies indicate higher prevalence in certain Asian populations, particularly in Taiwan and Japan, likely attributable to founder effects [16]. Despite its rarity, AADC-D represents a clinically significant disorder because of its severe lifelong disability and high morbidity [17]. The recent emergence of disease-modifying therapies, including gene therapy approaches targeting restoration of AADC function, has highlighted the critical need for early and accurate diagnosis to optimize treatment outcomes [2,17,18]. Evidence increasingly suggests that therapeutic efficacy is strongly associated with early intervention, underscoring the importance of reliable diagnostic strategies [8,9].

Current diagnostic approaches for AADC-D primarily rely on neurochemical analysis of cerebrospinal fluid (CSF), which typically demonstrates markedly reduced concentrations of dopamine and serotonin metabolites homovanillic acid (HVA) and 5-hydroxyindoleacetic acid (5-HIAA) [4,5,19,20,21]. Although informative, CSF analysis is invasive, technically demanding, and requires specialized laboratory expertise, limiting its accessibility in routine clinical settings. Alternative biochemical screening methods include measurement of 3-O-methyldopa (3-OMD) in dried blood spots using LC-MS/MS, which has enabled expanded newborn screening initiatives [13,22]. However, elevated 3-OMD concentrations are not entirely specific to AADC deficiency and may also occur in disorders affecting tetrahydrobiopterin (BH_4_) metabolism [3].

From a metabolomics perspective, direct measurement of AADC enzymatic activity offers a functional biomarker that reflects real-time pathway capacity and biochemical flux. Unlike static metabolite measurements, enzymatic activity assays provide dynamic insight into pathway functionality [3]. Traditionally, AADC enzyme activity has been determined by incubating biological samples with physiological substrates followed by quantification of reaction products using High-Performance Liquid Chromatography (HPLC) [4,6,19,20]. Despite its potential, plasma-based functional assessment remains underexplored as a minimally invasive metabolic biomarker for AADC-D diagnosis.

Given the critical importance of early detection and the limitations of current diagnostic methods, there is a need for reliable biochemical tools that complement existing molecular genetic testing and conventional neurochemical investigations. Plasma AADC activity measurement has the potential to provide a non-invasive functional assessment of enzyme activity that may support the biochemical evaluation of AADC deficiency.

Therefore, the objective of this study was to optimize and evaluate an HPLC-based assay for quantifying plasma AADC enzymatic activity using its physiological substrates, L-dopa and 5-hydroxytryptophan. Furthermore, the study aimed to assess its preliminary clinical application in healthy individuals and genetically confirmed AADC-deficient patients.

**Figure 1:**
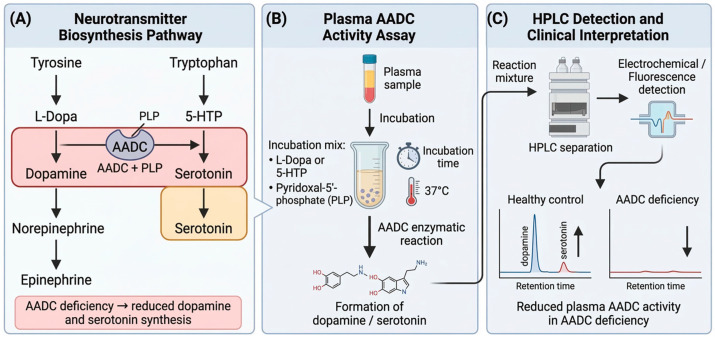
Principle of plasma AADC enzyme activity measurement. (**A**) Aromatic L-amino acid decarboxylase (AADC) catalyzes the decarboxylation of L-Dopa and 5-hydroxytryptophan (5-HTP) in the monoamine neurotransmitter pathways. (**B**) Experimental workflow: plasma is incubated with substrates and the cofactor pyridoxal-5′-phosphate (PLP). (**C**) Reaction products are quantified via HPLC. A reduction in product peaks serves as a functional biochemical biomarker for AADC deficiency.

The principle of plasma AADC enzymatic activity measurement and its application as a functional biochemical biomarker for AADC deficiency are illustrated in Figure 1.

## 2. Materials and Methods

### 2.1. Chemicals and Reagents

All chemicals and reagents used were of analytical grade. Dopamine hydrochloride and serotonin hydrochloride standards were obtained from Sigma-Aldrich, St. Louis, MO, USA. The enzymatic substrates L-3,4-dihydroxyphenylalanine (L-dopa) and 5-hydroxytryptophan (5-HTP) were also purchased from Sigma-Aldrich, St. Louis, MO, USA. Pyridoxal-5′-phosphate (PLP), used as an essential enzymatic cofactor, was also obtained from Sigma-Aldrich, St. Louis, MO, USA. To prevent analyte oxidation, reaction buffers were supplemented with dithioerythritol (DTE), L-ascorbic acid, and ethylenediaminetetraacetic acid (EDTA). Mobile phase components including potassium dihydrogen phosphate, sodium octanesulfonate, sodium perchlorate, and phosphoric acid were sourced from Merck, Darmstadt, Germany. All solvents, including methanol, acetonitrile, and water, were of HPLC grade.

### 2.2. Preparation of Standards and Reagents

A dopamine stock solution (0.5 mmol/L) was prepared in aqueous ascorbic acid solution and stored in aliquots at −80 °C. Calibration standards were freshly prepared prior to analysis by appropriate dilution in acidic aqueous medium. Working buffer solutions consisted of phosphate buffer (pH 7.0) containing reducing agents to maintain enzyme stability. Substrate solutions of L-dopa and 5-HTP were prepared fresh on the day of analysis. PLP cofactor solution was prepared in water and stored at 4 °C for a maximum of 8 h. Perchloric acid solutions were used for enzymatic reaction termination and protein precipitation.

### 2.3. Study Subjects and Sample Collection

This study was conducted in accordance with the Declaration of Helsinki and was approved by the Medical Research and Ethics Committee (MREC), Ministry of Health Malaysia (Approval No.: KKM/NIHSEC/P19-1733). Healthy control participants (*n* = 18) were recruited among adult laboratory personnel with no known history of inherited metabolic disorders, neurological disorders, or chronic illnesses. Inclusion criteria comprised healthy adults aged ≥18 years. Exclusion criteria included insufficient sample volume, hemolyzed specimens and samples with improper handling or storage conditions.

Blood samples from patients clinically suspected of AADC deficiency were obtained from participating hospitals under the approved study protocol. Patients were included if they were referred for biochemical and molecular evaluation of suspected AADC deficiency. Samples with insufficient volume, improper handling or unsuitable storage conditions were excluded from analysis to minimize enzymatic degradation.

Blood samples were collected by venipuncture into EDTA tubes and centrifuged at 4 °C to separate plasma. Plasma aliquots were stored at −80 °C until analysis.

### 2.4. Plasma AADC Enzyme Activity Assay

AADC enzyme activity was determined by measuring the enzymatic conversion of L-dopa to dopamine or 5-HTP to serotonin. Prior to substrate addition, plasma samples were pre-incubated with PLP to ensure complete saturation of the enzyme with its cofactor (PLP).

#### 2.4.1. L-Dopa Decarboxylation Assay

Plasma samples were pre-incubated with PLP solution and phosphate buffer for 120 min at 37 °C to ensure cofactor saturation of the enzyme. Based on optimization studies, a plasma volume of 120 µL and an L-dopa substrate concentration of 10 mM were used. The reaction was initiated by the addition of L-dopa and incubated for 120 min at 37 °C. The reaction was terminated by the addition of perchloric acid, followed by centrifugation to remove precipitated proteins. Supernatants were collected and stored at 4 °C for HPLC analysis.

#### 2.4.2. 5-HTP Decarboxylation Assay

Plasma samples were incubated with PLP and phosphate buffer containing reducing agents at 37 °C. Based on optimization studies, a plasma volume of 100 µL and a 5-HTP substrate concentration of 20 mM were used. The reaction was initiated by the addition of 5-HTP and incubated for 15 h at 37 °C. The reaction was terminated using perchloric acid, followed by centrifugation. Supernatants were collected for HPLC analysis. Blank reactions without substrate were included to correct for background signal.

### 2.5. HPLC Analysis

#### 2.5.1. Dopamine Detection

Dopamine was quantified using an Agilent HPLC system (Agilent Technologies, Santa Clara, CA, USA) equipped with electrochemical detection (ECD). Separation was achieved on a reversed-phase C18 column (4.6 × 150 mm, 5 µm particle size) using an isocratic mobile phase (phosphate buffer, ion-pairing reagent, EDTA, and methanol) at acidic pH. The flow rate was maintained at 1.0 mL/min, and the detector potential was optimized via voltammetric analysis.

#### 2.5.2. Serotonin Detection

Serotonin was analyzed using the same C18 column with fluorescence detection (FLD). The mobile phase consisted of citrate-phosphate buffer, ion-pairing reagent, EDTA, and methanol. Detection was performed at excitation and emission wavelengths of 278 nm and 325 nm, respectively.

### 2.6. Calculation of Enzyme Activity

Analyte concentrations were calculated by comparison of sample peak responses with calibration standards. Blank values were subtracted to obtain net product formation. AADC enzymatic activity was expressed as pmol/min/mL based on product concentration, incubation time, and dilution factors (Appendix A).

### 2.7. Method Validation

Analytical evaluation of the assay included optimization studies, linearity assessment, and intra-assay precision testing. Linearity was assessed across a concentration range of 5–5000 nM for dopamine and serotonin. Precision was evaluated by intra-assay variability using pooled plasma. Optimization studies of incubation conditions were performed to ensure assay reliability.

### 2.8. Optimization Study

The relationship between incubation time and plasma volume with dopamine and serotonin production was assessed by conducting plasma decarboxylation assays under varying incubation times (20 to 120 min for L-dopa; 2 to 20 h for 5-HTP) and volumes (20 to 120 µL).

### 2.9. Molecular Genetic Analysis

DNA was extracted from EDTA blood samples of two patients suspected of having AADC deficiency based on biochemical analysis using a magnetic bead-based protocol and subsequently subjected to Sanger sequencing of the 13 coding exons of the *DDC* gene (NM_001082971.2). Raw sequencing data were analyzed using SeqScape software v3.0 (Applied Biosystems, Waltham, MA, USA) to identify variants, and variant pathogenicity was predicted using Franklin and classified according to ACMG guidelines [17].

### 2.10. Ethics Approval

This study was approved by the Medical Research Ethics Committee, Ministry of Health (NMRR-19-196-46731) (KKM/NIHSEC/P19-1733). Written informed consent was obtained from all participants.

### 2.11. Statistical Analysis

Data were analyzed descriptively and are presented as mean values, coefficient of variation (CV), and reference intervals. Due to the limited number of positive cases available for analysis, no formal statistical comparisons between groups were performed.

## 3. Results

### 3.1. Method Validation

**Figure 2:**
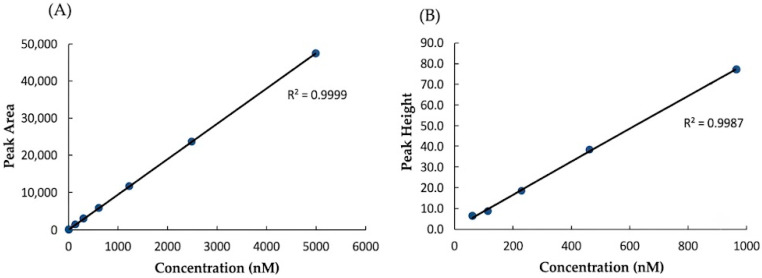
Linearity study or calibration curve for dopamine (**A**) and serotonin (**B**) of AADC activity assay. Dopamine was quantified by HPLC-ECD using peak area, while serotonin was quantified by HPLC-FLD using peak height.

Hydrodynamic voltammetry was performed to optimize detection sensitivity by measuring the dopamine peak height across E2 potentials from +50 mV to +450 mV. The optimum potential of +450 mV was achieved through a voltammogram study and was chosen for detection. Quantification of the samples was performed using an external standard of 1000 nM dopamine. A strong linear relationship was observed across the tested concentration range (5–5000 nM) for both dopamine and serotonin, indicating the method’s linearity. The method was found to be linear up to 5000 nmol/L, with a good linear relationship (R^2^) over the concentration range for L-dopa (R^2^ = 0.9999) and up to 1000 nmol/L for 5-HT (R^2^ = 0.9987) (Figure 2).

The plasma AADC enzyme activity assay was evaluated using L-dopa and 5-hydroxytryptophan (5-HTP) as physiological substrates. Assay precision was assessed using pooled plasma prepared from healthy adult samples (*n* = 15). For the L-dopa reaction, the intra-assay coefficient of variation (CV) was 8.81% at an enzyme activity of 15.33 pmol/min/mL. For the 5-HTP reaction, the intra-assay CV was 8.88% at an enzyme activity of 2.23 pmol/min/mL.

Plasma AADC activity was subsequently evaluated in individual healthy adult controls (*n* = 18). The mean plasma AADC activity was 29.3 pmol/min/mL for the L-dopa reaction, and 4.4 pmol/min/mL for the 5-HTP reaction. These values fell within the observed range of the healthy adult control group of 24–43 pmol/min/mL for L-dopa and 1.5–5.5 pmol/min/mL for 5-HTP respectively (Table 1). For comparison, the published pediatric reference interval for plasma AADC activity are 36–129 pmol/min/mL using L-dopa as substrate and 2.0–7.1 pmol/min/mL using 5-HTP as substrate.

**Figure 3:**
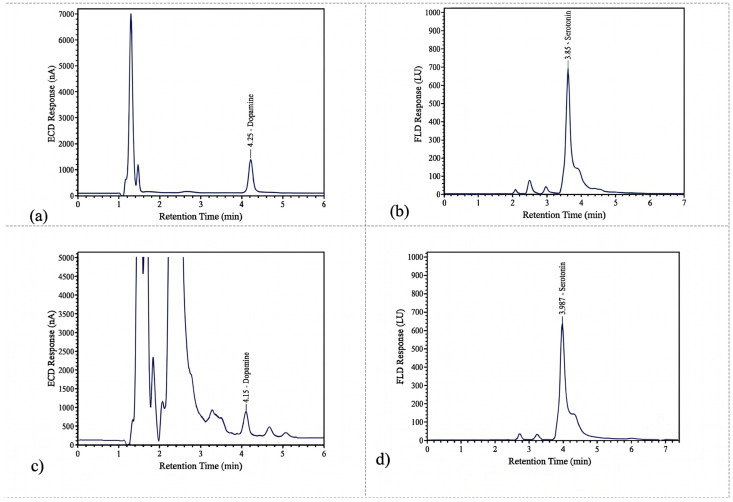
Chromatograms of dopamine and serotonin external standards and plasma sample for the AADC activity assay. HPLC analysis of (**a**) dopamine standard (1000 nM) by electrochemical detection (ECD), (**b**) serotonin standard (1000 nM) by fluorescence detection (FLD), (**c**) normal plasma sample following L-dopa decarboxylation and (**d**) normal plasma sample following 5-HTP decarboxylation. Retention times of the quantified analytes are indicated on each chromatogram.

Representative chromatograms of dopamine and serotonin standards and plasma samples obtained from the AADC activity assay are shown in Figure 3.

### 3.2. Optimization Study

Plasma L-dopa decarboxylation

The relationship between incubation time with substrate and dopamine formation was observed to be linear across the tested incubation times (10 to 120 min). The amount of L-dopa converted to dopamine by plasma AADC increased proportionally with increasing incubation period (Figure S1A, *R*^2^= 0.9448). An incubation time of 120 min was selected as the optimal condition to ensure sufficient dopamine formation in the reaction for reliable quantification. Similarly, the relationship between plasma volume with dopamine production demonstrated a linear trend across the tested volume (20 to 120 µL; *R*^2^ = 0.9987), indicating proportional increased in product formation with greater sample input (Figure S1). A plasma volume of 120 µL was selected as the optimal reaction volume for the subsequent assays.

Plasma 5-HTP decarboxylation

The relationship between incubation time with substrate and serotonin production was evaluated across a range of incubation periods from 2 to 20 h (Figure S2). An incubation time of 15 h was selected to enable adequate conversion of 5-hydroxytryptophan (5-HTP) to serotonin (5-HT) in the reaction (Figure S2). The relationship between plasma volume on serotonin production was also determined across the tested volume range (20 to 120 µL; Figure S2). A plasma volume of 100 µL was selected as optimal reaction volume for the subsequent assays. A final substrate concentration of 20 mM was used to allow for maximum activity for all subsequent assays.

For L-dopa decarboxylation (Figure S1), dopamine production increased linearly with incubation time up to 120 min, indicating that the enzyme remained catalytically active within this period. Similarly, an increase in plasma volume enhanced dopamine formation, with a strong linear correlation, suggesting that enzyme availability was not a limiting factor under the tested conditions. Based on these findings, an incubation time of 2 h and a plasma volume of 120 µL were selected as the optimal conditions to achieve sufficient dopamine production.

Conversely, the conversion of 5-HTP to 5-HT exhibited a different kinetic profile (Figure S2). While serotonin formation increased with plasma volume, it plateaued beyond 40 µL, suggesting a limit in product formation. Similarly, extending the incubation time beyond 15 h did not significantly enhance serotonin yield, indicating that prolonged incubation may not be beneficial. Serotonin production exhibited a non-linear trend, characterized by an initial increase in product concentration followed by fluctuations at later time points. This fluctuation pattern may reflect substrate depletion, enzyme instability, or possible feedback inhibition during prolonged incubation. As a result, an incubation time of 15 h and a plasma volume of 100 µL were selected as optimal conditions for serotonin production.

Optimization data are presented in Supplementary Figures S1 and S2.

### 3.3. Plasma AADC Activity in AADC-Deficient Patients

Table 2 presents the clinical features, biochemical and molecular profiles, and enzymatic activity results of the studied patients. The biochemical analysis of Patient 1 (1.1 years old) and Patient 2 (3 years old) showed normal pterins and normal urine biogenic amines level with abnormally low 5-HIAA/HVA ratio compared to the reference values. Both patients also had normal blood phenylalanine concentrations, 32.4 (18.0–180.0 µmol/L) for Patient 1 and 34.7 (18.0–180.0 µmol/L) for Patient 2. However, both presented with hypotonia and developmental delay leading the clinician to suspect an underlying neurotransmitter disorder. The AADC enzyme activity assay in plasma demonstrated reduced enzymatic activity in both patients compared to the reference range.

Using L-dopa as substrate, mean enzyme activity in healthy controls was 29.3 pmol/min/mL (range 24–43). Patient 1 demonstrated markedly reduced activity (12.4 pmol/min/mL), while Patient 2 showed reduced activity at the lower limit of the reference range (26.1 pmol/min/mL). Using 5-HTP as substrate, the mean activity in controls was 4.4 pmol/min/mL (range 1.5–5.5). Patient 1 exhibited reduced activity (1.5 pmol/min/mL), whereas Patient 2 demonstrated activity within the observed healthy control range (5.1 pmol/min/mL).

Overall, Patient 1 demonstrated markedly reduced enzyme activity using both substrates, whereas Patient 2 showed lower L-dopa decarboxylation activity with 5-HTP activity within the observed control range. The biochemical findings were interpreted together with the clinical presentation and molecular genetic results. The results demonstrated a lower enzyme activity in the AADC-deficient patients compared with healthy controls. These findings are consistent with AADC deficiency.

### 3.4. Genetic Analysis

Genetic analysis confirmed the presence of pathogenic variants in the *DDC* gene in both studied patients (Figure S3). Patient 1 harbored two heterozygous variants at c.175G>A p.(Asp59Asn) in exon 2 and c.714+4A>T p.(?) in intron 6. Patient 2 was found to harbor a homozygous intronic variant, c.714+4A>T. These molecular findings were consistent with the markedly reduced plasma AADC enzymatic activity observed in both patients and supported the biochemical diagnosis of AADC deficiency. Representative Sanger sequencing electropherograms are presented in Supplementary Figure S3.

## 4. Discussion

The present study demonstrates that measurement of plasma Aromatic L-Amino Acid Decarboxylase (AADC) activity provides a functional biochemical marker capable of distinguishing genetically confirmed AADC-deficient patients from healthy individuals. Both patients exhibited reduced L-dopa decarboxylation activity compared with the published reference interval, supporting the potential diagnostic utility of direct enzyme activity measurement. Because AADC catalyzes the final step in monoamine biosynthesis, reduced plasma activity, particularly with L-dopa as substrate, may reflect impaired monoamine neurotransmitter biosynthesis associated with AADC deficiency [7,23].

Currently, molecular genetic testing remains the primary confirmatory method for diagnosing AADC deficiency [23]; however, it may be associated with high cost and longer turnaround times. Cerebrospinal fluid (CSF) neurotransmitter analysis can also aid diagnosis but requires invasive lumbar puncture and specialized laboratory expertise [6,9,23]. Consensus guidelines recognize reduced plasma AADC enzyme activity as one of the core diagnostic indicators of AADC deficiency [23]. Therefore, a minimally invasive plasma-based biochemical assay that directly assesses enzyme function offers a valuable complementary diagnostic approach. This present study highlights the utility of plasma AADC activity measurement as a functional biochemical complement to molecular genetic testing through the implementation and evaluation of an HPLC-based assay in our laboratory setting and its preliminary clinical application in genetically confirmed AADC-deficient patients.

In this study, plasma AADC activity was measured using both physiological substrates, L-dopa and 5-HTP. Consistent with previous reports [1,8,23], greater product formation was observed with L-dopa compared with 5-HTP under the optimized assay conditions. Furthermore, L-dopa substrate analysis showed greater difference in enzyme activity between the affected patients and healthy controls, suggesting that catecholamine pathway assessment may be more sensitive for detecting functional AADC impairment in plasma.

Optimization studies demonstrated that appropriate incubation time, plasma volume, and substrate concentration were important for achieving reliable product formation and assay performance. These conditions were subsequently applied for plasma AADC activity measurement in healthy controls and AADC-deficient patients.

The biochemical findings were concordant with molecular genetic results. Patient 1 harbored two heterozygous pathogenic variants in the *DDC* gene, whereas Patient 2 was homozygous for the intronic variant c.714+4A>T, a variant frequently reported in Asian populations [4]. Franklin analysis predicted c.175G>A as a likely pathogenic while c.714+4A>T variant as a pathogenic. The concurrence of reduced plasma AADC activity and pathogenic *DDC* variants supports the validity of enzyme activity measurement as a functional correlate of genetic deficiency.

Although Patient 2 demonstrated 5-HTP decarboxylation activity within the observed healthy control range, the diagnosis of AADC deficiency was supported by reduced L-dopa decarboxylation activity together with molecular confirmation of a homozygous pathogenic DDC variant. This observation suggests that L-dopa decarboxylation may provide greater discriminatory value than 5-HTP in the assessment of plasma AADC activity consistent with previous reports [1,8].

Previous studies have consistently demonstrated markedly reduced or undetectable plasma AADC activity in affected patients [1,4,6]. Our findings are in agreement with these reports and further support the diagnostic relevance of plasma enzyme activity measurement. In contrast, urinary dopamine and related metabolites have been shown to be unreliable diagnostic markers due to variable renal AADC expression and residual enzymatic activity [9,23]. Direct plasma enzyme quantification, therefore, provides a more specific assessment of systemic AADC catalytic capacity.

It should be noted that the published reference intervals used for comparison were established in pediatric populations, whereas the healthy control samples analyzed in the present study consisted of healthy adults and were included primarily for method evaluation purposes rather than formal reference interval establishment. Consequently, interpretation of patient enzyme activity was based principally on comparison with the published pediatric reference intervals and the corresponding molecular genetic findings.

This study has limitations. The number of confirmed patients analyzed was small due to the rarity of AADC deficiency. Consequently, findings should be interpreted descriptively rather than inferentially. In addition, the analytical evaluation focused primarily on assay optimization, linearity, and intra-assay precision. Further studies are required to assess additional validation parameters, including accuracy, stability, carryover and inter-laboratory reproducibility. Larger multicenter studies are required to further evaluate diagnostic performance and potential application in broader screening strategies.

In conclusion, plasma AADC activity measurement by HPLC shows potential as a functional biochemical biomarker reflecting monoamine biosynthetic capacity. The findings of this study support the potential utility of this minimally invasive assay as a complementary tool to genetic testing and existing neurochemical analyses in the biochemical diagnosis of aromatic L-amino acid decarboxylase deficiency.

## 5. Conclusions

This study demonstrates that plasma Aromatic L-Amino Acid Decarboxylase (AADC) activity can be reliably quantified using a validated HPLC-based assay and demonstrates potential as a functional biochemical biomarker for AADC deficiency. Markedly reduced L-dopa decarboxylation activity was observed in genetically confirmed patients compared with published reference intervals, supporting the potential diagnostic utility of direct enzyme activity measurement.

Among the two physiological substrates evaluated, L-dopa provided greater product formation and demonstrated greater differentiation of enzyme activity values between affected individuals and healthy controls, making it the preferred substrate for plasma AADC activity assessment.

The concordance between reduced plasma enzyme activity and pathogenic DDC variants highlights the utility of integrating functional biochemical testing with molecular genetic analysis. Plasma AADC activity measurement may therefore serve as a complementary diagnostic tool alongside existing cerebrospinal fluid and genetic investigations.

Further studies involving larger patient cohorts are required to further evaluate the diagnostic performance and broader clinical applicability of this approach.

## Figures and Tables

**Table 1:** Validation and optimization parameters of the plasma AADC activity assay using L-dopa and 5-HTP substrates.

Parameters	Substrate	
	L-Dopa	5-HTP
**Linearity (R^2^)**	0.9999	0.9987
**Upper Limit of Linearity (nM)**	5000	1000
* Precision (CV) %	8.81 (15.3)	8.88 (2.2)
**Incubation Time (hours)**	2 h	15 h
**Plasma Volume (µL)**	120	100
**Healthy Adult Controls,** **Mean Activity (pmol/min/mL)**	29.3	4.4

* Precision data are expressed as coefficients of variation (CV, %). Values in parentheses represent the mean plasma AADC activity (pmol/min/mL) of pooled plasma used for intra-assay precision assessment.

**Table 2:** Clinical Characteristics, Plasma AADC Activity, and Genetic Findings in AADC-Deficient Patients.

Parameter	Patient 1	Patient 2	Reference Range
Sex (M/F)	Male	Female	—
Age at Diagnosis (years)	1.1	3.0	—
Plasma AADC Activity			
L-Dopa (pmol/min/mL)	12.4	26.1	36–129
5-HTP (pmol/min/mL)	1.5	5.1	2.0–7.1
Clinical Features	Hypotonia, developmental delay	Hypotonia, developmental delay	—
Genetic Findings (*DDC* gene)	c.175G>A (p.Asp59Asn) p.(?); c.714+4A>T (two heterozygous variants)	c.714+4A>T (homozygous intronic variant)	—

## Data Availability

The original contributions presented in this study are included in the article/Supplementary Materials. Further inquiries can be directed to the corresponding author.

## Supplementary Materials

The following supporting information can be downloaded at: https://www.mdpi.com/article/10.3390/metabo16070444/s1, Figure S1: Optimization of plasma AADC activity assay using L-dopa as substrate. Relationship between (A) incubation time and dopamine concentration and (B) plasma volume and dopamine concentration. Figure S2: Optimization of plasma AADC activity assay using 5-hydroxytryptophan (5-HTP) as substrate. Relationship between (A) incubation time and serotonin concentration and (B) plasma volume and serotonin concentration. Figure S3: Representative Sanger sequencing electropherograms of pathogenic *DDC* variants identified in the studied patients. (A) Missense variant c.175G>A p.(Asp59Asn) and p.(?) (B) Intronic splice-site variant c.714+4A>T.

## Abbreviations

The following abbreviations are used in this manuscript:

3-OMD	3-methyldopa
5-HIAA	5-hydroxyindoleacetic acid
5-HTP	5-hydroxytryptophan
AADC	Aromatic L-amino acid decarboxylase
CSF	Cerebrospinal fluid
HPLC	High-Performance Liquid Chromatography
HVA	Homovanillic acid
IEM	Inborn error of metabolism
MREC	Medical Research and Ethics Committee
NIH	National Institute of Health
L-DOPA	L-3,4-dihydroxyphenylalanine
PLP	Pyridoxal-5′-phosphate

## Appendix A. Calculation of AADC Enzyme Activity

AADC activity was calculated and expressed as pmol/min/mL of plasma using the following formula:

AADC activity = (Final Sample Conc. (nM) × Dilution Factor) Incubation Time (min)

These results were expressed as pmol/min/mL equivalent to mU/L, where 1 mU = 1 nmol/min/L. 1 nM = 1 pmol/mL; no additional unit conversion was required.
