# Supplementary material for: Plasma Aromatic L-Amino Acid Decarboxylase Activity by HPLC as a Functional Biomarker for the Diagnosis of Aromatic L-Amino Acid Decarboxylase Deficiency
Source: Metabolites. 2026 Jun 25;16(7):444. doi: 10.3390/metabo16070444 (PMC13414192; doi:10.3390/metabo16070444)
Supplement: Supplementary file 1 [file metabolites-16-00444-s001.zip › metabolites-4350717-supplementary.pdf]

(A)

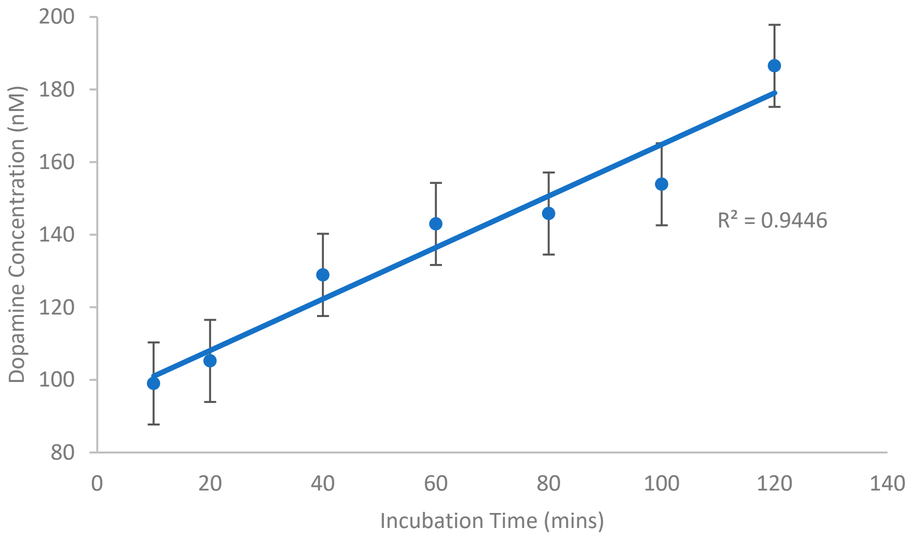

(B)

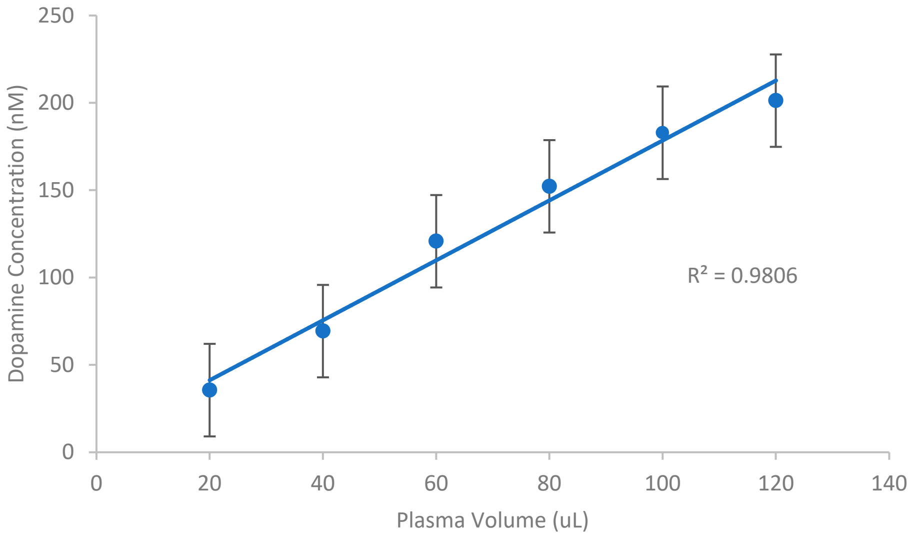

Figure S1: Optimization of plasma AADC activity assay using L-dopa as substrate. Relationship between (A) incubation time and dopamine concentration and (B) plasma volume and dopamine concentration.

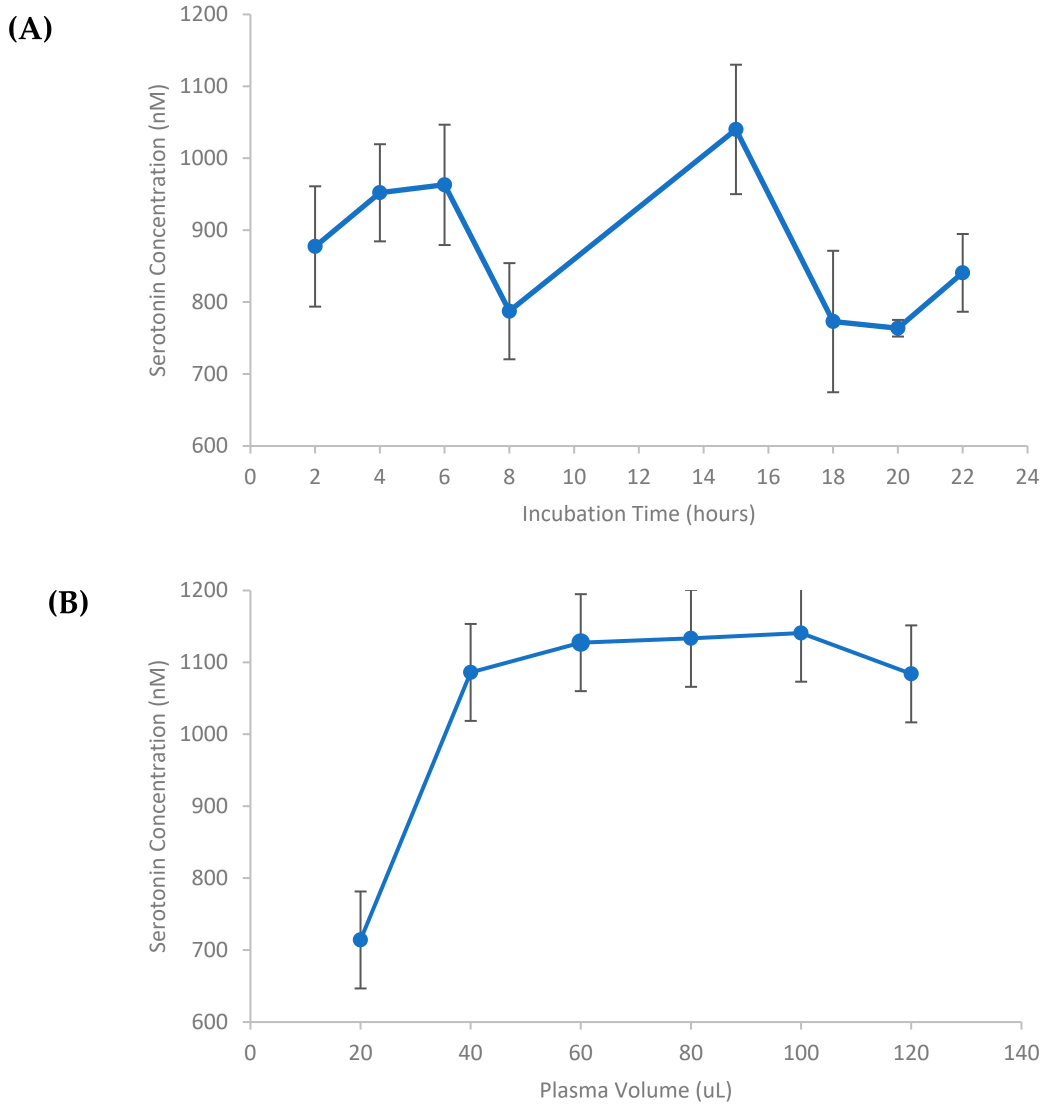

Figure S2: Optimization of plasma AADC activity assay using 5-hydroxytryptophan (5-HTP) as substrate. Relationship between (A) incubation time and serotonin concentration and (B) plasma volume and serotonin concentration

(A) (B)

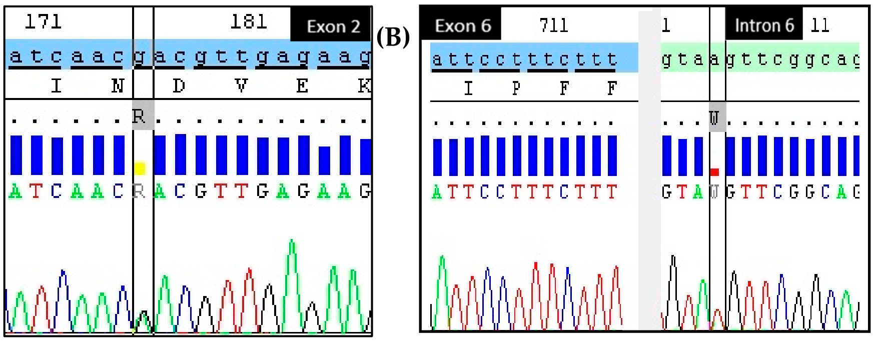

Figure S3. Representative Sanger sequencing electropherogram of pathogenic *DDC* gene variants identified in the studied patients. (A) Missense variant c.175G>A p.(Asp59Asn) and p.(?) (B) Intronic splice variant c.714+4A>T.
